# Responding to the weather: energy budgeting by a small mammal in the wild

**DOI:** 10.1093/cz/zoz023

**Published:** 2019-05-17

**Authors:** Taylor Hume, Fritz Geiser, Shannon E Currie, Gerhard Körtner, Clare Stawski

**Affiliations:** 1 Centre for Behavioural and Physiological Ecology, Zoology, University of New England, Armidale, NSW, 2351, Australia; 2 Leibniz Institute for Zoo and Wildlife Research, Alfred-Kowalke-Str. 17, Berlin, 10315, Germany; 3 Department of Biology, Norwegian University of Science and Technology, Trondheim, 7491, Norway

**Keywords:** antechinus, heterothermy, marsupial, semelparous, temperature, torpor

## Abstract

Energy conservation is paramount for small mammals because of their small size, large surface area to volume ratio, and the resultant high heat loss to the environment. To survive on limited food resources and to fuel their expensive metabolism during activity, many small mammals employ daily torpor to reduce energy expenditure during the rest phase. We hypothesized that a small terrestrial semelparous marsupial, the brown antechinus *Antechinus stuartii*, would maximize activity when foraging conditions were favorable to gain fat reserves before their intense breeding period, but would increase torpor use when conditions were poor to conserve these fat reserves. Female antechinus were trapped and implanted with small temperature-sensitive radio transmitters to record body temperature and to quantify torpor expression and activity patterns in the wild. Most antechinus used torpor at least once per day over the entire study period. Total daily torpor use increased and mean daily body temperature decreased significantly with a reduction in minimum ambient temperature. Interestingly, antechinus employed less torpor on days with more rain and decreasing barometric pressure. In contrast to torpor expression, activity was directly related to ambient temperature and inversely related to barometric pressure. Our results reveal that antechinus use a flexible combination of physiology and behavior that can be adjusted to manage their energy budget according to weather variables.

The ability of endothermic mammals to maintain and regulate a constant high body temperature (*T*_b_) that is largely independent from ambient temperature (*T*_a_) provides many advantages, including the capability to be active at night and throughout all seasons (Scholander et al. 1950; Tattersall et al. 2012; Withers et al. 2016). However, in small mammals, this can be problematic because their surface area to volume ratio is greater than large mammals. As heat loss is a function of both the relative surface area and the *T*_b_ − *T*_a_ differential, small mammals use proportionally more energy for thermoregulation at low *T*_a_, and therefore have higher metabolic demands relative to their body mass than large mammals when maintaining a high and constant *T*_b_ (Tattersall et al. 2012; Withers et al. 2016).

To be able to cope with these energetic costs, many small mammals employ torpor, during which their metabolic rate, *T*_b_, heart rate, and other physiological functions are substantially reduced (Ruf and Geiser 2015). Torpor is often expressed to conserve energy in response to adverse environmental conditions, such as low *T*_a_, food reduction, and heavy rainfall (Boyer and Barnes 1999; Ruf and Geiser 2015). Torpor is also crucial for survival in response to sudden changes in the environment, such as fires and storms (Nowack et al. 2015; Stawski et al. 2015; Geiser et al. 2018). Furthermore, by lowering energy demands, torpor use can allow individuals to reduce foraging time, which may reduce predation and starvation risk (Stawski and Geiser 2010; Hanna and Cardillo 2014; Turbill and Stojanovski 2018).

Torpor expression fluctuates with changes in resource availability, competition, risks of predation, and the costs of dedicating energy to foraging. Animals may therefore trade-off time spent active by employing torpor to avoid these problems if they outweigh the benefits gained by foraging (Stawski and Geiser 2010; Levy et al. 2012). This ability to identify risks is important for small mammals to respond appropriately to current and approaching weather conditions; deciding whether it would be more beneficial for them to forage or to employ torpor on any given day. Endotherms are able to sense changes in *T*_a_ using primary sensory neurons through the periphery in their skin (Glotzbach and Heller 1976). Some placental mammals can sense changes in barometric pressure, which often precedes rainfall or inclement weather that may interrupt food availability (Vickery and Bider 1981; Czenze and Willis 2015); however, data for marsupials are not available.

To determine how small mammals respond to, or predict, variations in weather patterns, we quantified torpor expression and activity patterns of free ranging female brown antechinus *Antechinus stuartii*, during the Austral winter in relation to *T*_a_, precipitation and changes in barometric pressure. Brown antechinus are small heterothermic insectivorous marsupials from the family Dasyuridae and are distributed along the eastern coast and ranges of Australia (Geiser 1988). They are short-lived marsupials that undergo an almost complete yearly population turnover (Woolley 1966; McAllan and Dickman 1986). Males die shortly after expending excessive energy during a brief 2-week mating period in late winter that leads to starvation and increased parasite loads, whereas most females die after their young have been weaned (Woolley 1966); however, one-third of females may survive to reproduce for a second breeding season (Rojas et al. 2014). We hypothesized that torpor would be employed extensively by females leading up to the energetically demanding reproductive period as they are typically food-limited at this time (Dickman 1989; Stawski and Rojas 2016). Furthermore, we predicted that torpor expression would be greater in response to challenging environmental conditions, such as low *T*_a_ and rainfall.

## Material and Methods

Our study site was located at Guy Fawkes River National Park, NSW, Australia (30°04′58.6″S, 152°20′0.9″E); the work was conducted between 18 May and 27 June 2015 during the austral autumn/winter. This time period is ∼2–3 months before the 2-week breeding period that occurs during August (known from trapping survey data conducted throughout the year at the study site). Permits from the NSW National Parks and Wildlife Services and the University of New England animal ethics committee (AEC13-088) were obtained before commencing research. *T*_a_ was recorded every 10 min using a temperature data logger (Thermochron, DS 1921 G, Maxim, resolution 0.5°C, Rio Robles, CA, USA) tied to a small branch in the shade ∼1 m above the ground in the study site. Rainfall and barometric pressure variables were obtained from a NSW National Parks and Wildlife Service weather station positioned ∼2 km from the field site.

We used aluminum box traps (Elliott Scientific Equipment, Upwey, Australia) to capture a total of 17 female *A. stuartii*. The traps were baited with a mixture of peanut butter, oats, and honey and insulated with polyester fiber to prevent hypothermia during overnight trapping. During the afternoon, we setup 9 trap lines of 25 traps (a total of 225 traps each night). We set each trap ∼4–5 m apart and checked them early the next morning. Captured study animals were later transported to the University of New England (130 km and ∼2 h drive) where they were weighed to the nearest 0.1 g using electronic scales (mean capture body mass: 23.4 ± 2.2 g, *n = *17) and housed individually in cages over night from the day of capture. Individuals were implanted with a temperature-sensitive transmitter with a unique frequency for animal identification (2.0–2.3 g, Sirtrack, Havelock North, New Zealand) under isoflurane/oxygen anesthesia as described by Rojas et al. (2010). Transmitter pulse rate as a function of temperature was calibrated to the nearest 0.1°C between 15°C and 40°C, at 5°C increments in a temperature-controlled water bath. Prior to implantation, we coated each transmitter in wax (Paraffin/Elvax, Minimitter, Bend, Oregon, USA) and ensured that the transmitter was <10% of body mass as recommended for small terrestrial animals (Rojas et al. 2010). The transmitters and surgical instruments were sterilized prior to the surgery in 70% alcohol. For surgery, a small part of ventral fur was removed using a razor blade and the bare skin was cleaned and sterilized before a small incision was made and the transmitter was inserted. The incision was sutured (muscle and skin separately) using coated Vicryl (3.0 metric, Ethicon Inc Sommerville, New Jersey, USA.) after which it was sprayed with a local anesthetic (Xylocaine, AstraZeneca Pty Ltd, North Ryde, NSW, Australia) and spray bandage (BSN Medical (Aust) Pty Ltd, Clayton, VIC, Australia). The reproductive age of the animals was recorded at time of surgery, however, behavior and physiology between first and second reproductive year females did not differ. For post-surgery recovery, the antechinus were kept in a warm room and fed cat food, meal worms and water before being released at the site of capture the next day.

To locate each individual, we radio-tracked the antechinus each morning over the initial 40 days, and then once a week for a further 30 days using receivers (ICOM IC-R10, Icom Inc. Osaka, Japan) and Yagi antennae (Sirtrack, Havelock North, New Zealand). Overall, antechinus were tracked for a mean of 47.7 ± 12.5 days depending on the battery life of the transmitter (range of 28–69 days).

During radio-tracking, manual *T*_b_ readings using a stopwatch were recorded immediately when a signal was detected to ensure immediate data collection of *T*_b_ before data loggers were deployed. Receiver/loggers with antennas were setup near nest sites to record transmitter pulse intervals every 10 min. Each device was equipped with a scanner receiver, logger and a 12 V battery (Körtner and Geiser 2000) that was sealed in a plastic container and wrapped in a plastic bag. H-frame antennae were tied to a tree in a position to maximize signal detection. The batteries were exchanged once a week and the pulse rate data were downloaded. Pulse rates were converted to *T*_b_ using a second-order polynomial equation (*r*^2^ > 0.999) based on the individual transmitter calibration data.

We defined torpor as *T*_b_ ≤ 31.5°C, as calculated on the basis of Willis (2007; Equation 4: *T*_b-onset_ – 1 SE *=* (0.041)BM + (0.040)*T*_a_ + 31.092; SE *=* standard error, BM = body mass, *T*_a_ = mean daily *T*_a_ for study period) to enable the detection of shallow torpor bouts. Torpor bout duration was calculated as the time *T*_b_ remained below the torpor threshold for durations >30 min. As antechinus often displayed more than 1 torpor bout/day (maximum of 5 torpor bouts), the total daily torpor (TDT) use in minutes for each study day was calculated for each animal from sunrise to sunrise (data from the Australian Bureau of Meteorology) and was used for analyses. The duration of activity bouts was calculated from the time the individual was out of the range of the receiver/logger (reception range ∼5–10 m), indicating that they had left the nest, until the time they returned. For analyses, we used the total daily activity (TDA) duration summed for each day of the study period between sunrise and sunrise. Only the days that an individual left and returned to the same nest were included. However, it is possible that during longer activity bouts the animal may have used another nest location for a short period of time.

Statistical analyses were conducted using R (Version 3.4.3, 2018). A *P* < 0.05 was considered significant and means are presented with ± standard deviation (SD). We ran a Pearson’s correlation test on the weather variables and as all had a correlation statistic <0.7 (i.e., Henning et al. 2005) we included all of the weather variables and first-order interaction terms (barometric pressure × *T*_a min_ = −0.32; barometric pressure × rain = −0.04; *T*_a min_ × rain = 0.26). Linear mixed effects models were fitted (package “nlme”; Pinheiro et al. 2018) to determine the effect of *T*_a min_ (°C), rainfall (mm) and absolute barometric pressure (hPa) on the measured variables (TDT [min], daily mean *T*_b_ [°C] while resting at the nest [*T*_b mean_], TDA [min]). Body mass (g) at capture was included as a covariate and multiple measures for individuals by including individual (ID) as a random effect. We tested for normality (Q–Q plot) and homoscedasticity (residual plot). For the initial model, we included interactions between the weather variables. The best-fit model was determined using Akaike information criterion (AIC; Akaike 1974) and performing a log likelihood ratio test on the top 2 models (package “MuMIn”; Bartoń 2013). Linear mixed effects models were also fitted to determine if the measured variables differed on days of changing barometric pressure. The change in barometric pressure was categorized as increasing (1–11.9 hPa), decreasing (−1 to −10.4 hPa), or null (−0.9 to 0.9 hPa), by subtracting each day’s barometric pressure from the previous day. Following this, a post hoc Tukey test (package “multcomp”; Hothorn et al. 2008) was employed to determine the difference.

## Results

The *T*_a_ throughout the study period ranged from a minimum of −1.5°C to a maximum of 18.0°C and the mean was 7.5°C ± 3.6°C (*n = *62; Table 1). Rainfall (mm) was recorded on 25 of the total 70 study days with a mean daily rainfall of 1.6 ± 4.2 mm (*n = *62; Table 1). A total of 115.8 mm of rain fell throughout the entire study period and the maximum amount of rain that fell on a single day was 24.0 mm.

**Table 1.** Summary of weather variables including means with SD and absolute minimum and maximum values recorded

Variable	Mean	Absolute minimum	Absolute maximum
*T*_a_ (°C)	7.5 ± 3.6	−1.5	18.0
*T*_a max_ (°C)	12.8 ± 2.3	6	18.0
*T*_a min_ (°C)	3.6 ± 2.9	−1.5	12.0
Rainfall (mm)	1.6 ± 4.2	0.0	24.0

**Figure 1.**
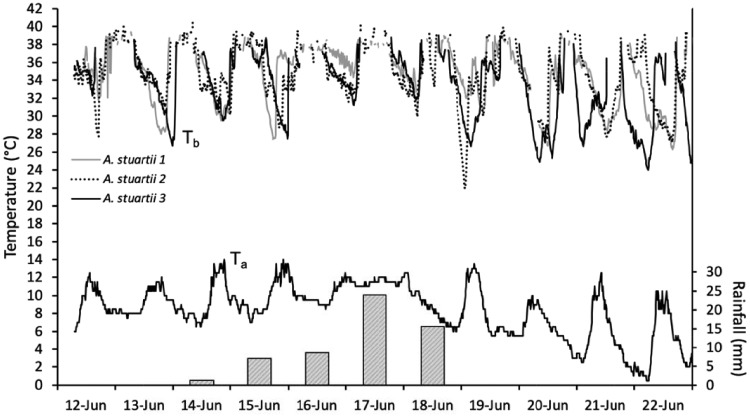
Body temperature (*T*_b_), ambient temperature (*T*_a_), and rainfall data over 11 days of the study period. The *T*_b_ of 3 individual antechinus are represented by different lines and shown by the key, *T*_a_ by a solid black line, and rainfall by bar plots.

**Figure 2.**
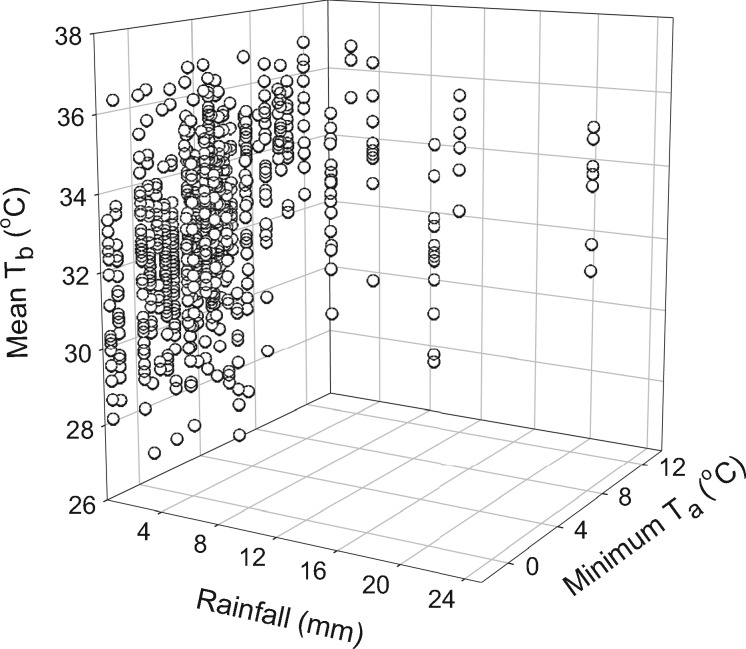
3D scatterplot of daily mean resting body temperature (*T*_b mean_) as a function of daily minimum ambient temperature (*T*_a min_) and rainfall.

The mean daily *T*_b mean_ varied with *T*_a_ throughout the study period (Figure 1) and for all individuals was 32.6°C ± 0.8°C (*n = *17, *N = *793), with an absolute lowest *T*_b min_ of 18.1°C and an absolute highest *T*_b max_ of 40.9°C. Our best-fit model (Table 2) included *T*_a min_, rainfall, barometric pressure, and the interaction between *T*_a min_ and rainfall. Therefore, the effect of *T*_a min_ was mediated by rain, such that *T*_b mean_ was lowest on cold and dry days (Figure 2). In addition, *T*_b mean_ increased with lower barometric pressure and was therefore significantly higher on days of falling barometric pressure (*z* = −4.3, *P *<* *0.0001; *n = *17, *N = *793; Table 3).

**Table 2.** The 4 best-fit models with variables (minimum ambient temperature = *T*_a min_ [°C], rainfall = rain [mm], absolute barometric pressure = bp [hPa], body mass = bm [g]) that explain differences in TDT use (min), mean daily body temperature (*T*_b mean_, °C), and TDA duration (min)

Variable	Model	AICc	Delta	*P*
*T*_b mean_	***T*_a min_ + rain + bp + bm + *T*_a min_ × rain**	**2899.9**	**0.00**	**0.005**
	*T*_a min_ + rain + bp + bm	2901.3	1.35	
	*T*_a min_ + rain + bp + bm + *T*_a min_**×** rain + *T*_a min_**×** bp	2909.6	9.65	
	*T*_a min_ + rain + bp + bm + *T*_a min_**×** rain + *T*_a min_**×** bp + rain **×** bp	2920.9	21.02	
TDT	***T*_a min_ + rain + bp + bm + *T*_a min_ × rain**	**10488.5**	**0.00**	**0.264**
	*T*_a min_ + rain + bp + bm + *T*_a min_**×** rain + *T*_a min_**×** bp	10489.3	0.81	
	*T*_a min_ + rain + bp + bm + *T*_a min_**×** rain + *T*_a min_**×** bp + rain **×** bp	10489.9	1.34	
	*T*_a min_ + rain + bp + bm	10492.9	**4.40**	
TDA	***T*_a min_ + rain + bp + bm**	**10917.7**	**0.00**	**0.156**
	*T*_a min_ + rain + bp + bm + *T*_a min_**×** rain	10917.7	0.03	
	*T*_a min_ + rain + bp + bm + *T*_a min_**×** rain + *T*_a min_**×** bp	10918.5	0.81	
	*T*_a min_ + rain + bp + bm + *T*_a min_**×** rain + *T*_a min_**×** bp + rain **×** bp	10918.9	1.20	

The *P-*value is from a log likelihood ratio test performed for the 2 top models. The best-fit model for each variable is indicated in bold.

**Table 3.** Means with SD of TDT use, mean daily body temperature (*T*_b mean_), and TDA duration on days of decreasing and increasing barometric pressure (BP)

Variable	Decreasing BP	Increasing BP	*P*
*T*_b mean_ (°C)	32.9 ± 0.7	32.2 ± 0.9	**<0.0001**
TDT (min)	291.9 ± 80.2	373.5 ± 121.2	**<0.0001**
TDA (min)	595.1 ± 99.7	502.7 ± 101.5	**<0.0001**

Bold *P*-value indicates a significant difference.

**Figure 3.**
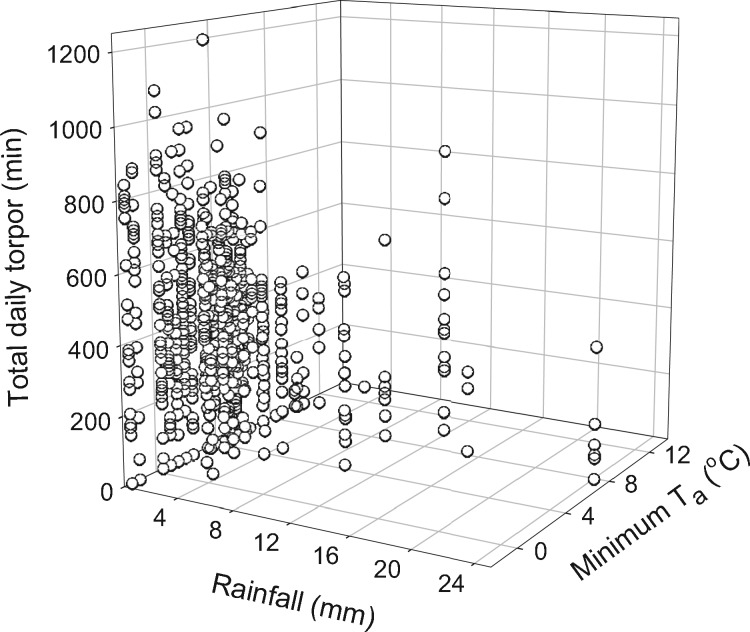
3D scatterplot of TDT use as a function of daily minimum ambient temperature (*T*_a min_) and rainfall.

Torpor was expressed by all antechinus studied and torpor use was recorded on 82.2 ± 11.6% (*n = *17) of study days. The mean TDT use throughout the whole study period (including days of torpor and no torpor) was 330.8 ± 100.9 min (*n = *17, *N = *793); the maximum TDT during a single day was 1,200 min when average *T*_a_ was 7.4°C. The best-fit model for TDT (Table 2) revealed that TDT was affected also by *T*_a min_, rainfall, barometric pressure, and the interaction between *T*_a min_ and rainfall. This interaction term revealed that TDT was longer on cold and dry days (Figure 3). A change in barometric pressure also significantly affected the TDT; on days of falling barometric pressure, less torpor was employed (*z* = 4.3, *P *<* *0.0001; *n = *17, *N = *793; Table 3).

**Figure 4.**
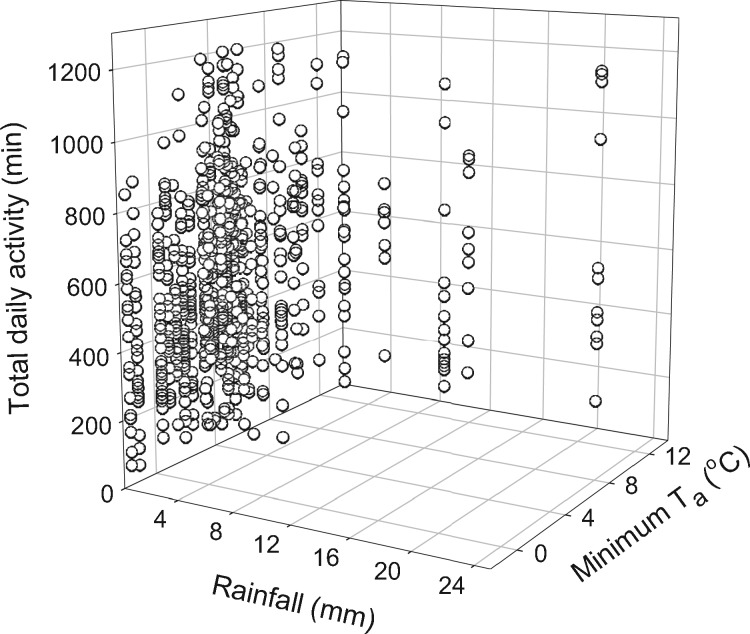
3D scatterplot of TDA duration as a function of daily minimum ambient temperature (*T*_a min_) and rainfall.

The mean TDA duration (sunrise to sunrise) was 549.7 ± 96.3 min (*n = *17, *N = *794), ranging from 40 to 1,220 min. Opposite to torpor use, activity increased at higher *T*_a_ (Figure 4). The best-fit model (Table 2) for TDA also included rainfall and barometric pressure. However, the best-fit model for TDA differed from TDT and *T*_b mean_ and did not include the interaction between *T*_a min_ and rainfall. Conversely to TDT, the amount of time antechinus were active throughout the day was greater on days of falling barometric pressure (*z* = −3.4, *P *<* *0.0001; *n = *17, *N = *794; Table 3).

## Discussion

Our study revealed that thermal and foraging biology of free ranging *A. stuartii*, 2–3 months before the breeding period, were affected by prevailing weather conditions. Torpor and activity were linked in female antechinus as torpor use was negatively correlated whereas activity was positively correlated with *T*_a_. These results support previous observations that torpor and activity are governed by changes in the environment (Rojas et al. 2014; Stawski et al. 2015). Interestingly, antechinus in our study employed more torpor during periods of less rain and increasing barometric pressure. Therefore, our results suggest that physiological and behavioral flexibility is an important aspect of an individual’s response to sudden changes in the environment, such as inclement weather.

Torpor was used almost daily (on 82.2 ± 11.6% of all study days) by all female *A. stuartii* during the study period. Torpor expression increased at lower *T*_a_, resulting in lower daily *T*_b mean_. Furthermore, and somewhat unexpected, antechinus spent less time torpid when it was raining and at low and decreasing barometric pressure. We recorded rainfall on 25 of the total 70 study days with 9 of these days recording >5.0 mm of rainfall. During such periods of prolonged rainfall, foraging returns would likely be limited and heat loss would increase, especially if the fur is wet (Withers et al. 2016). Therefore, one might assume that the animals would employ longer and deeper torpor during the rain event, but this was not the case. Instead, the longest and the deepest bouts of torpor occurred on a day that had been preceded by 4 days of significant rainfall (>5.0 mm; Figure 1).

TDA duration was longer at higher *T*_a_s and decreasing barometric pressure. This may, in part, explain the torpor patterns we recorded. It is likely to be profitable for antechinus to forage during the warmer conditions preceding a weather front as insect activity is typically higher (Pellegrino et al. 2013; McFarlane et al. 2015; Dagaeff et al. 2016). Kaspari and Weiser (2000) found that ants were more active during wetter seasons that promote humid microclimates and the release of nutrients as result of moist leaf litter (McColl 1975). Furthermore, some rodents increase activity during rainfall if the temperature is higher (Vickery and Bider 1981). Rainy days were warmer and antechinus often forage under thick ground cover to likely avoid getting wet. In addition, cloudless nights with high barometric pressure will be colder and antechinus will likely have reduced foraging opportunities suggesting that it is more beneficial for antechinus to decrease activity and conserve energy by using torpor. This observation suggests that antechinus begin to employ more torpor and reduce activity as barometric pressure increases and reduce torpor with decreasing barometric pressure that often signals inclement weather. Therefore, the results of our study indicate that antechinus are more energy restricted in response to cold conditions rather than rain alone.

Other species such as some bats and birds appear to perceive and in some cases predict changing weather patterns through sensing barometric changes, but the method by which species can predict these changes is still poorly understood (Heupel et al. 2003; Wikramanayake et al. 2006; Breuner et al. 2013; Bender and Hartman 2015; Streby et al. 2015). Changes in barometric pressure usually foreshadow changes in *T*_a_ and other weather variables (Breuner et al. 2013). Therefore, an individual’s ability to detect changes in barometric pressure and predict the associated weather patterns would allow it to precisely manage its energy budget and/or take refuge during a storm. A laboratory study showed that a decline in barometric pressure stimulated an increase in food intake in birds, suggesting they were preparing for a storm (Breuner et al. 2015). This is particularly important for insectivorous animals as many insects respond to changes in barometric pressure by altering their activity and mating behaviors (Pellegrino et al. 2013; McFarlane et al. 2015; Dagaeff et al. 2016). Indeed, insectivorous bats appear to be able to track changes in barometric pressure and adjust their activity accordingly (Dechmann et al. 2017), and it is likely that antechinus can do the same.

Limited data suggest that the paratympanic organ of the middle ear in vertebrates may function as a sensor for changes in barometric pressure (von Bartheld and Giannessi 2011). To date, this organ has primarily been found and described not only in birds, but also in 1 bat species (von Bartheld and Giannessi 2011). We were unable to find any literature on marsupials with regard to this organ, but it has been suggested that some mammals may be able to use the hair cells of the inner ear to detect pressure changes (Funakubo et al. 2010). Therefore, more research is needed to determine the mechanism by which marsupials may be able to detect changes in barometric pressure, the sensitivity of this mechanism, and if they are able to predict weather patterns using it.

Overall, our study suggests that *A. stuartii* alter torpor expression and activity according to prevailing and oncoming environmental conditions which may enhance their survival during weather changes. Being able to predict and respond to changes in the weather provides many survival benefits for small mammals like antechinus, primarily by balancing foraging and fat accumulation when conditions are favorable and employing torpor during adverse weather for energy conservation. This is particularly important for antechinus that have a single breeding period, as it is likely they attempt to build up fat reserves before the energy demanding reproductive period in late winter and early spring.
